# Artificial vision: the effectiveness of the OrCam in patients with advanced inherited retinal dystrophies

**DOI:** 10.1111/aos.15001

**Published:** 2021-09-26

**Authors:** Xuan‐Thanh‐An Nguyen, Jan Koopman, Maria M. van Genderen, Henk L.M. Stam, Camiel J.F. Boon

**Affiliations:** ^1^ Department of Ophthalmology Leiden University Medical Center Leiden The Netherlands; ^2^ Royal Dutch Visio, Centre of Expertise for Blind and Partially Sighted People Amsterdam The Netherlands; ^3^ Department of Ophthalmology University Medical Center Utrecht Utrecht The Netherlands; ^4^ Bartiméus, Diagnostic Center for Complex Visual Disorders Zeist The Netherlands; ^5^ Department of Ophthalmology Amsterdam UMC Academic Medical Center Amsterdam The Netherlands

**Keywords:** cone‐rod dystrophies, low vision, OrCam, quality of life, retinitis pigmentosa, visual aids

## Abstract

**Purpose:**

To investigate the impact of the OrCam MyEye 2.0 (OrCam) on the quality of life and rehabilitation needs in patients with advanced retinitis pigmentosa (RP) or cone‐rod dystrophies (CRD). The OrCam is a wearable low‐vision aid that converts visual information to auditive feedback (e.g. text‐to‐speech, barcode and facial recognition).

**Methods:**

Patients with a clinical diagnosis of RP (*n* = 9, 45%) or CRD (*n* = 11; 55%), and a best‐corrected visual acuity of ≤20/400 Snellen were invited to participate in this study. Questionnaires were administered at baseline and after 5.2 (standard deviation ± 1.5) weeks, which included the Dutch version of the National Eye Institute Visual Functioning Questionnaire (NEI‐VFQ), the Participation and Activity Inventory (PAI) and the OrCam Function Questionnaire (OFQ).

**Results:**

Following OrCam testing, significant improvements were observed in the ‘near activities’ subscale of the NEI‐VFQ (p < 0.001); the ‘visual functioning’ subscale of the re‐engineered NEI‐VFQ (p = 0.001); the ‘reading’ rehabilitation goal of the PAI (p = 0.005) and the overall score of the OFQ (p < 0.001). The observed changes in questionnaire scores did not differ between phenotypes. Advantages and limitations of the OrCam were reported by patients. Three patients (15%) continued rehabilitation with the OrCam after completion of this study.

**Conclusions:**

The OrCam mainly improves reading domains in patients with advanced stages of RP or CRD. Further improvements in the OrCam are needed to address current limitations, which may enhance its utility for patients with RP or CRD.

## Introduction

Inherited retinal dystrophies (IRDs) comprise a diverse group of rare eye diseases characterized by progressive loss of photoreceptor function, ultimately leading to severe visual impairment (Cremers et al. 2018). Inherited retinal dystrophies (IRDs) can be differentiated, in part, through the order of which cells are lost (Cremers et al. 2018). In retinitis pigmentosa (RP), degeneration of rods precedes that of cones, resulting in initial symptoms of nyctalopia and peripheral visual field loss (Hamel 2006; Hartong et al. 2006; Ferrari et al. 2011). Ultimately, central vision is also lost. Conversely, in cone‐rod dystrophies (CRD), the process of photoreceptor degeneration follows the opposite sequence of events than in RP, causing predominant symptoms of central vision loss, photophobia and colour vision impairment followed by peripheral vision loss and night blindness in later stages of the disease (Hamel 2007; Thiadens et al. 2011). Loss of visual function due to RP or CRD has detrimental effects on a patient’s well‐being and on their ability to perform daily activities, although the extent and areas of difficulties may vary between these phenotypes (Latham et al. 2015).

For most patients with IRDs, the visual prognosis remains poor, as curative treatments are unavailable or are still under investigation. Therefore, emphasis should be on assisting patients with managing their disease, for example, through low‐vision rehabilitation services (Lamoureux et al. 2007). The goal of low‐vision rehabilitation is not to restore vision, but to utilize residual vision to its maximum potential (Langelaan et al. 2009). This may be achieved by low‐vision centres through the prescription of low‐vision aids (LVAs), ranging from (non‐)optical aids to electronic assistive technologies. The selection of appropriate LVAs for an individual patient is complex, and several factors need to be considered prior to prescription, such as a patient’s visual and cognitive ability, disease stage, occupation and own rehabilitation goals (Das et al. 2019; Lorenzini & Wittich 2020).

The OrCam MyEye (https://www.orcam.com), or OrCam in short, is a relatively recent addition to the list of commercially available LVAs. The OrCam is a portable LVA that can be attached to the frame of a patient’s eyeglasses. It contains a small camera that converts digital or printed text to real‐time auditive feedback using optical character recognition technology. As such, the intended audience for the OrCam consists of severe visually impaired or blind patients that have lost the ability to read independently. Aside from text‐to‐speech capabilities, the OrCam also contains colour, object, barcode, money and facial recognition. Thus, the OrCam has the potential to improve the performance of multiple daily activities in visually impaired patients. However, the impact of a single LVA remains unclear, as low‐vision rehabilitation programmes typically offer multiple LVAs and multidisciplinary services over the course of rehabilitation. This makes it difficult to distinguish the contribution of a single device or service on a patient’s rehabilitation progress (Moisseiev & Mannis 2016; Waisbourd et al. 2019). Insights into the effectiveness of the OrCam will provide knowledge on which patients are most likely to benefit from the device and will also inform us on which daily activities may improve when using devices such as the OrCam. In addition, as the target of interest has to be within the OrCam’s field of view, we also investigated whether the feasibility of the OrCam differed in those with different visual abilities, for example, patients with peripheral blindness or central blindness. For this purpose, this study investigated the effectiveness of the OrCam on the quality of life and the perceived difficulties in daily activities in severe visually impaired or blind patients caused by either RP or CRD.

## Methods

### Participants

Patients that were scheduled for one of the two Dutch low‐vision rehabilitation centres, Bartiméus (Amsterdam, the Netherlands) or Royal Dutch Visio (Amsterdam, the Netherlands), were invited to participate in this study. Inclusion criteria for this study were a clinical diagnosis of RP or CRD based on full‐field electroretinography data, and a best‐corrected visual acuity (BCVA) of 20/200 Snellen acuity or worse. An additional inclusion criterion for patients with RP was a constricted peripheral visual field on Goldmann kinetic perimetry (<20° around point of fixation using a V4e stimulus) at the most recent examination, whereas for patients with CRD, an absolute central scotoma with residual peripheral fields was present in all. Identification of a causative gene was not a requirement for this study. Exclusion criteria for this study included the presence of other ocular diseases, significant cognitive impairment, insufficient understanding of the Dutch language and tremor‐inducing conditions that could impede gesture recognition by the OrCam (e.g. Parkinson’s disease). Ethical approval for this study was obtained from the Medical Ethics Committee at the Leiden University Medical Center. The study adhered to the tenets of the Declaration of Helsinki, and informed consent was signed by all participants.

### OrCam study protocol

**Fig. 1:**
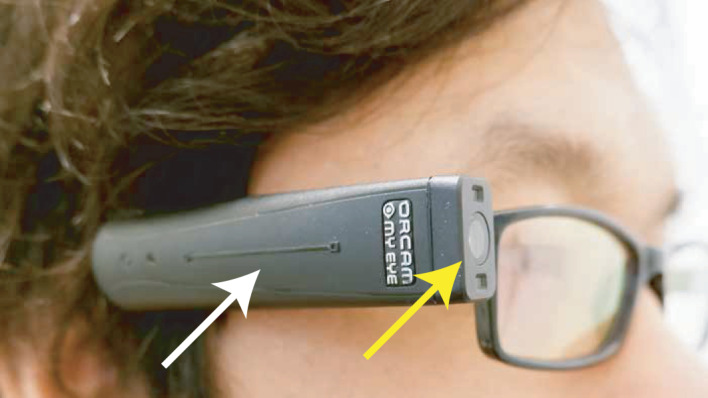
OrCam MyEye 2.0 is a portable low‐vision aid that can be mounted to the arms of a pair of glasses. The processor unit has an internal speaker, charge port, power button and a touch bar for activation and menu navigation (white arrow). Furthermore, the OrCam contains an optical sensor (yellow arrow), that returns scanned text or objects to auditive feedback via the internal speaker or through a Bluetooth connected earpiece. A mini flashlight is also present to aid in lower light situations. In addition to text‐to‐speech functions, the OrCam also contains colour (selective) barcodes, money, person and object recognition features. In order for person and object recognition features to function, it is required to scan the desired target in advance, subsequently storing this information in the internal memory of the OrCam. The OrCam is activated via the touch bar, or hands‐free via automatic target recognition, or by performing gesturing motions (e.g. pointing at a target).

Questionnaires were administered in patients using a personal interview‐format at initial visit and at follow‐up (mean follow‐up: 5.2 weeks ± standard deviation [SD] 1.5). Additionally, patients underwent visual acuity testing using a Snellen letter chart and received instructions on the OrCam at first visit. Both centres followed a similar OrCam instruction protocol, performed by experienced instructors, to ensure identical training between centres. Different models of the OrCam exist, which differ in price and their available features (https://www.orcam.com). For this study, the OrCam MyEye 2.0 was tested by all patients (Fig. 1), and instructions were given on the following functions: text recognition, facial recognition, barcode recognition, object recognition, money recognition, colour recognition and telling time (Moisseiev & Mannis 2016).

The OrCam’s features are activated by pressing the touch bar located on the device itself; or hands‐free via automatic target recognition, or by performing gesturing motions (e.g. pointing at an object for recognition features or flicking the wrist for time telling functions). After receiving detailed instructions, patients were lent the OrCam for personal use without any restrictions. Patients were called after approximately 1 week to assess whether they required changes in personal settings, or if any technical difficulties with the OrCam were encountered. At follow‐up, patients returned the OrCam and the same questionnaires as at baseline were administered. Optionally, patients were able to share their overall experience with the OrCam using an open‐ended question format. Remarks on the (dis)advantages of the OrCam that were mentioned by ≥25% of the cohort were included in the results.

### Questionnaires

Three questionnaires were used in this study, which included the National Eye Institute Visual Function Questionnaire (NEI‐VFQ), the Participation and Activity Inventory (PAI), and the OrCam Function Questionnaire (OFQ). Patients were instructed to answer all questionnaires as if they were using their own LVAs, with the addition of the OrCam as a LVA at follow‐up assessment.

The NEI‐VFQ is a 25‐item questionnaire with 14 supplemental items and is one of the most common vision‐related quality of life questionnaires used in ophthalmic research. The NEI‐VFQ is designed to evaluate aspects of daily living, which can be categorized into 12 different subscales (Mangione et al. 2001). For our study, the driving subscale was omitted, as none of the patients were permitted to drive. Answers given by patients were subsequently recoded into a 100‐point scale, where a higher score represents better (visual) functioning, as suggested by the original authors (Mangione et al. 2001). An overall composite score was calculated by averaging the scores of all subscales, whilst excluding the ‘general health’ subscale.

The PAI, formerly known as the Dutch Activity Inventory, is a validated questionnaire that is used in Dutch low‐vision rehabilitation centres to systematically assess the rehabilitation goals of patients (Bruijning et al. 2010a; Bruijning et al. 2010b; Elsman et al. 2018; Macnaughton et al. 2019). The PAI is based on the Activity Inventory designed by Massof and colleagues (Massof et al. 2007), which was modified in order to extend to the European population (Bruijning et al. 2010a; Bruijning et al. 2010b; Latham et al. 2015). For this study, a shortened version of the PAI was used, which included 11 rehabilitation goals related to central or peripheral vision (Table S1) (Latham et al. 2015). Patients were instructed to rate each goal on two aspects: importance and difficulty. Importance is rated on a Likert scale ranging from 0 (not important) to 3 (very important), whereas the difficulty scale goes from 0 (not difficult) to 4 (impossible). Subsequently, a priority score is calculated as the product of importance and difficulty for each included goal. The maximum achievable priority score is 12, with a higher priority score signifying a greater rehabilitation need for this specific rehabilitation goal.

The OFQ is a non‐validated questionnaire that was developed solely for this study. The questionnaire contained 14 items regarding vision‐related daily activities. The OFQ uses a 5‐level Likert scale, with possible difficulty scores being 1 (no difficulty), 2 (some difficulty), 3 (moderate difficulty), 4 (very difficult) or 5 (impossible due to disease). The activities included on the OFQ are as follows:

1. Reading a newspaper or book.
2. Reading for longer than 30 min without getting tired.
3. Reading an e‐mail.
4. Reading text from a distant sign such as a street sign.
5. Reading handwritten text.
6. Identifying different money bills.
7. Recognizing colours on clothing pieces.
8. Recognizing familiar objects, such as your keys or phone, at home.
9. Recognizing a familiar product in the grocery store.
10. Finding your way in the grocery store.
11. Reading a product label.
12. Recognizing familiar faces at home.
13. Recognizing familiar faces within an unfamiliar environment
14. Telling time

### Rasch analysis

Rasch analysis was performed exploratively on the NEI‐VFQ and OFQ using the Andrich rating scale model (Winsteps 4.6.0) (Massof & Fletcher 2001; Stelmack et al. 2002; Pesudovs et al. 2010). Rasch analysis converts ordinal scores into an interval scale and provides patient’s ability and item difficulty using logit values for the underlying construct. In our study, patients with higher (visual) ability and items of greater difficulty are placed more negatively of the logit scale, whereas more positive logit values reflect patients with lower (visual) ability and items with less difficulty. For NEI‐VFQ, re‐engineering of the questionnaire was guided by previous authors, who proposed a two subscale structure: visual functioning and socio‐emotional subscales (Table S1) (Stelmack et al. 2002; Pesudovs et al. 2010). For the OFQ, three items were removed to fit Rasch analysis, demonstrating reliable person and item separation values (reliability >0.8), scale targeting (difference between mean item and person measures <1.0 logit) and unidimensionality (variance accounted by the principal component >60%) (Table S1). Changes in person measures after OrCam rehabilitation were assessed using a stacked analysis (Anselmi et al. 2015).

### Statistical analysis

Data were analysed using the SPSS version 25.0 (IBM Corp, Armonk, NY, USA). Visual acuity data were converted to Logarithm of the Minimum Angle of Resolution (logMAR) values. For hand movement vision, light perception vision and no light perception, logMAR values of 2.7, 2.8 and 2.9 were used, respectively (Talib et al. 2017). Best‐corrected visual acuity (BCVA) in the better‐seeing eye of included patients were categorized into two groups: severe visual impairment (SVI; 20/400 ≤ BCVA < 20/200) or blindness (BCVA < 20/400), based on criteria set by the World Health Organization (World Health Organization 2019). As data were normally distributed, a paired 2‐tailed *t* test was used to determine significant changes in raw scores for each instrument. The effect of age, vision categories (SVI or blindness) and phenotypes (RP or CRD) on the likelihood of change were also investigated using a linear mixed model. A p‐value of 0.05 or less was considered clinical significant, and correction for multiple testing using the Bonferroni method was applied where appropriate.

## Results

Clinical characteristics of the patients are presented in Table 1. Twenty patients with IRD were enrolled in the study, of which nine patients were clinically diagnosed with RP (45%), and 11 patients with CRD (55%). Patients had an average BCVA of 1.5 logMAR (SD ± 0.4), which is equivalent to 20/640 Snellen visual acuity. Aside from visual field patterns, there were no differences in clinical characteristics between the two phenotypes. All patients had previously undergone low‐vision rehabilitation, and the majority of patients (*n* = 19; 95%) included in this study were in possession of at least one LVA with text‐to‐speech capabilities (Table 1).

**Table 1:** Clinical characteristics and prescribed visual aids in patients of this cohort.

Variable	Total (*n* = 20)	Retinitis pigmentosa (*n* = 9)	Cone‐rod dystrophies (*n* = 11)	p‐value
Age in years (mean ± SD)	47.6 ± 16.3	51.3 ± 16.5	44.5 ± 16.2	0.366
Male (*n*, %)	12 (60%)	7 (78%)	5 (45%)	0.197
Disease duration in years (mean ± SD)*	30.8 ± 12.8	33.5 ± 13.6	28.6 ± 12.3	0.406
Follow‐up in weeks (mean ± SD)	5.2 ± 1.5	5.0 ± 0.9	5.3 ± 1.9	0.634
logMAR BCVA (mean ± SD)	1.5 ± 0.4	1.5 ± 0.4	1.5 ± 0.5	0.881
Visual impairment (*n*, %)				
Severe impairment	9 (45%)	4 (44%)	5 (45%)	0.999
Blindness	11 (55%)	5 (56%)	6 (55%)	
Visual field pattern				
Central island	9 (45%)	9 (100%)	0 (0%)	<0.001
Central scotoma with peripheral remnants	11 (55%)	0 (0%)	11 (100%)	
Optical aids (*n*, %)				
Glasses	13 (65%)	6 (67%)	7 (64%)	
Telescopes	3 (15%)	1 (11%)	2 (18%)	
Hand or stand magnifiers	9 (45%)	3 (33%)	6 (55%)	
Non‐optical aids (*n*, %)				
Filter glasses	11 (55%)	6 (67%)	5 (46%)	
Illumination control	8 (40%)	4 (44%)	4 (36%)	
Braille	6 (30%)	2 (22%)	4 (36%)	
White cane	13 (65%)	8 (89%)	5 (46%)	
Text‐to‐speech products (*n*, %)^†^				
Screen reading software	14 (70%)	7 (56%)	9 (82%)	
Daisy reader (physical or digital)	14 (70%)	7 (78%)	7 (64%)	
Text‐to‐speech mobile applications	16 (80%)	8 (89%)	8(73%)	

p‐Values were derived from the independent *t*‐test, *χ* ^2^ test or Fisher’s exact test.

BCVA = best‐corrected visual acuity, logMAR = Logarithm of the Minimum Angle of Resolution, SD = standard deviation.

* Disease duration was defined as the difference between age at baseline and age at first symptom onset.

^†^ Text‐to‐speech products included software (e.g. JAWS, SuperNova, Window Eyes, VoiceOver), equipment, and mobile applications that convert digital or printed text to auditive feedback (e.g. Seeing AI or KNFB reader).

### National Eye Institute Visual Function Questionnaire

**Fig. 2:**
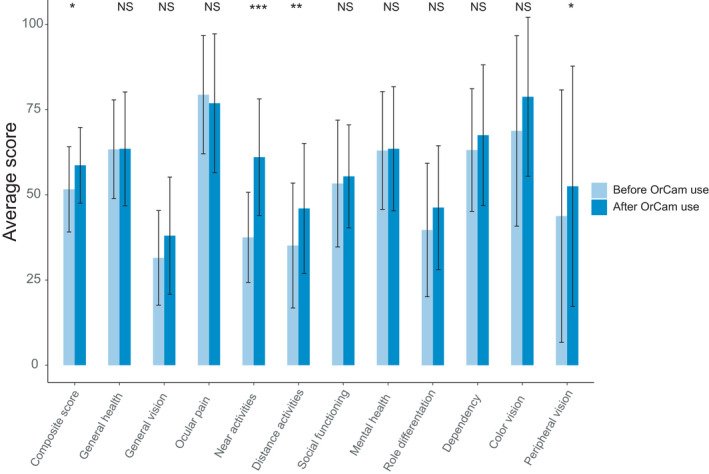
Average scores on the subscales of the National Eye Institute Visual Functioning pre‐ and postrehabilitation with the OrCam. The bar heights represent the mean scores of each subscale, and the black error bars indicate the corresponding standard deviation. Higher scores indicate better functional performance. Critical value of significance was set at 0.004 following correction for multiple testing (0.05/11). NS = not significant; *p < 0.05; **p < 0.01; ***p < 0.001.

At initial visit, the NEI‐VFQ showed a significantly lower score on the peripheral vision subscale in patients with RP compared to patients with CRD (p = 0.014). Other subscales on the NEI‐VFQ were found to be comparable between subgroups, including the overall composite score (Table S2). Rasch analysis revealed mean person measures of 0.53 (SD ± 0.64) and −0.18 (SD ± 0.59) logits for the visual functioning and socio‐emotional subscales, respectively. At follow‐up, significant improvements were observed in the raw scores of the near activities’ subscale (+23.5, 95% CI: 13.2–33.9; p < 0.001), which was not found for other subscales after correction for multiple testing (adjusted p‐value = 0.004; Fig. 2). The observed change was not affected by phenotype (p = 0.798), initial age (p = 0.089) or vision classification (p = 0.317). A significant change was also observed on the Rasch‐calibrated visual functioning subscale, showing an improvement of −0.65 logits (95% CI: −0.97 to −0.32; p = 0.001) after OrCam use. No significant change was found in the socio‐emotional subscale (−0.14, 95% CI: −0.40 to 0.11; p = 0.257) after rehabilitation.

### Participation and Activity Inventory Questionnaire

A summary of the priority scores for each goal on the PAI is provided in Table 2. Goals with the highest priority scores, indicating goals with the highest rehabilitation needs, were ‘mobility indoors within an unfamiliar environment’ and ‘personal administration’ for patients with RP; whereas the highest priority scores were found in the ‘reading’ and ‘personal administration’ goals for patients with CRD (Table 2). Whilst the order of priority for rehabilitation goals differed between phenotypes, there was no significant difference in average scores for each goal (Table S2). Bivariate analysis revealed a correlation between the priority score of the ‘mobility indoors within an unfamiliar environment’ goal and age at initial visit (*r* = 0.570; p = 0.009), suggesting that the rehabilitation need for the ‘mobility indoors within an unfamiliar environment’ goal becomes greater with increasing age.

**Table 2:** Priority scores as measured on the Participation and Activity Inventory questionnaire in patients with retinitis pigmentosa and cone‐rod dystrophies.

Retinitis pigmentosa		Cone‐rod dystrophies	
Rehabilitation goal	Priority score	Rehabilitation goal	Priority score
Mobility indoors within an unfamiliar environment	6.6 ± 4.8	Reading	7.1 ± 3.4
Personal administration	6.0 ± 4.0	Personal administration	7.0 ± 3.7
Grocery shopping	5.4 ± 4.0	Grocery shopping	5.4 ± 4.2
Public transportation	5.2 ± 3.0	Mobility indoors within an unfamiliar environment	5.1 ± 2.5
Reading	5.2 ± 3.0	Computer use	4.5 ± 2.0
Writing	4.6 ± 5.1	Public transportation	4.2 ± 3.2
Mobility outdoors	4.4 ± 4.0	Mobility outside	4.0 ± 3.1
Computer use	3.7 ± 3.6	Writing	3.1 ± 2.5
Recognition and communication	2.3 ± 2.9	Mobility indoors at home	2.5 ± 2.9
Mobility indoors at home	1.7 ± 2.6	Keeping time and following a schedule	1.9 ± 2.8
Keeping time and following a schedule	0.7 ± 2.0	Recognition and communication	1.5 ± 2.1

Rehabilitation goals for patients with retinitis pigmentosa or cone‐rod dystrophies are shown in descending order of priority. Priority scores are shown as means ± standard deviation. The maximum achievable priority score was 12, indicating a goal with the highest rehabilitation need.

Out of the 11 rehabilitation goals included, ‘reading’ was the only goal that improved after rehabilitation with the OrCam, as shown as a lower priority score at follow‐up (−2.6, 95% CI: −4.2 to −0.9; p = 0.005). When analysing the underlying tasks of the ‘reading’ goal, a significant lower priority score was found for the task ‘reading ordinary‐sized print’ (−3.9, 95% CI: −6.4 to 1.3; p = 0.005), which was not found for other tasks related to the ‘reading’ goal.

### OrCam Function Questionnaire

**Fig. 3:**
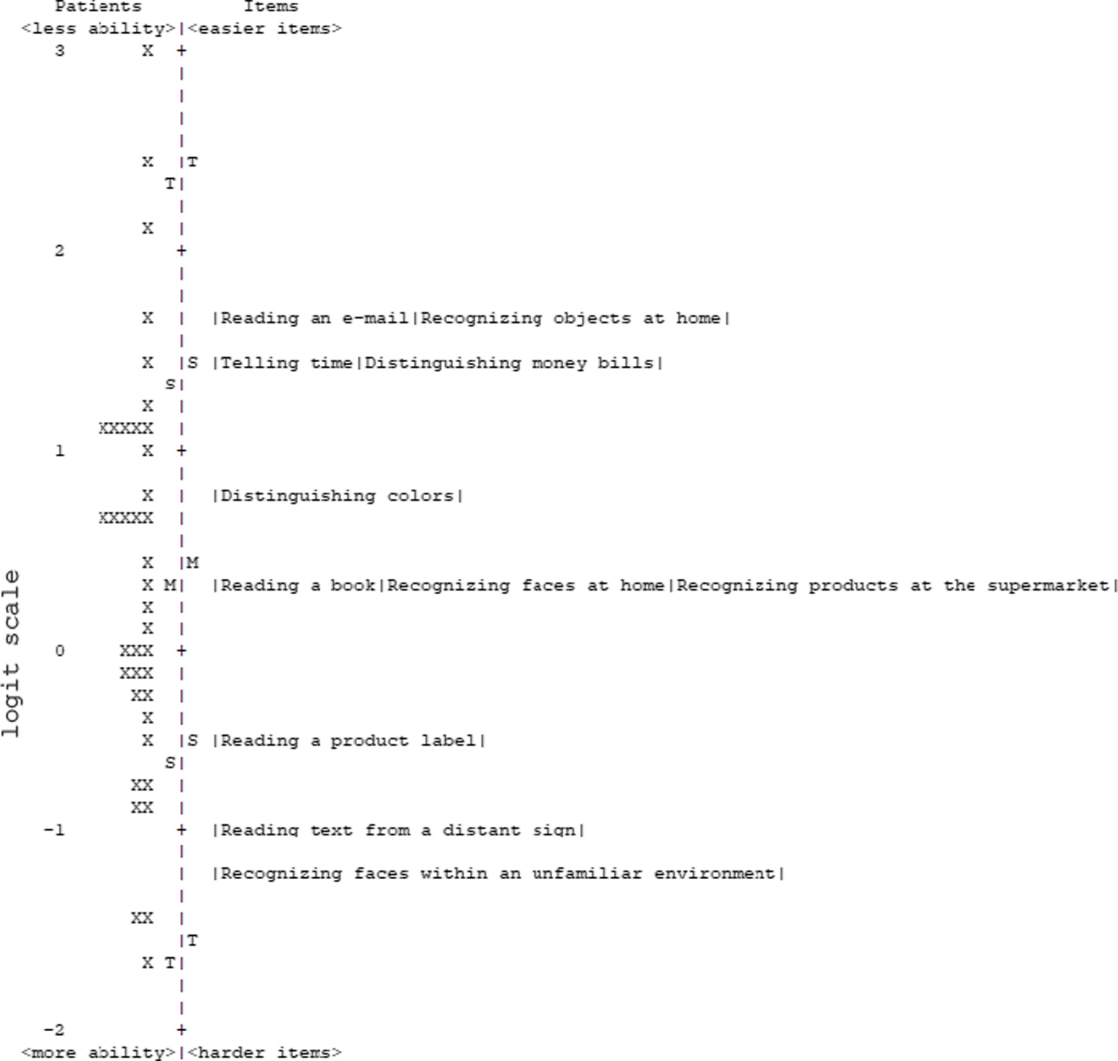
Stacked person‐item map of the OFQ questionnaire. Patients are shown as crosses and are mapped across the vertical line based on their (visual) ability measured in logits. For context, a patient with high abilities (i.e. no difficulty in performing activities) would be placed at the bottom of the logit scale. Similarly, item are also mapped according to their measure in logits, with the hardest items placed at the bottom of the scale. M, mean; S, 1 standard deviation from the mean; T, 2 standard deviations from the mean.

An item‐person map based on Rasch analysis of the OFQ questionnaire is shown in Figure 3. Items on the OFQ that were considered most difficult for this cohort were: ‘recognizing familiar faces within an unfamiliar environment’ (−1.67 logits)’, reading text from a distant sign’ (−1.40 logits), and ‘reading a product label’ (−0.90 logit); whereas ‘reading an e‐mail’ (1.18 logits) and ‘recognizing familiar objects at home’(1.15 logits) were considered the least difficult tasks. The average person measure was 0.43 logits (SD ± 0.92), which improved significantly following OrCam rehabilitation (−1.11, 95% CI: −1.61 to 0.61; p < 0.001). The observed change did not differ between phenotypes (p = 0.696).

### Overall experience with the OrCam

At final visit, patients shared their overall experience with the OrCam. Fifteen patients (75%) reported that the OrCam’s text recognition features functioned well in optimal light conditions. However, these features were less reliable in poorly lighted or dark rooms. Object and facial recognition features were not tested by most patients (*n* = 16; 80%), as patients reported that the current study period was too short to adequately test these features, or they did not consider these features necessary for their daily activities. Main advantages and limitations of the OrCam MyEye 2.0 provided by this cohort are summarized in Table 3. After completion of this study, two patients with RP (10%; aged 24 and 60) and one patient with CRD (5%; aged 51) continued with rehabilitation with the OrCam. The remaining patients (*n* = 17; 85%) did not resume rehabilitation with the OrCam. Reasons for not continuing with the OrCam, that were mentioned by at least five patients, were: (1) having text‐to‐speech products with similar functions as the OrCam (e.g. Seeing AI or KNFB reader); (2) pricing of the OrCam; (3) and lack of features that were considered important to a patient (e.g. assistance with navigation). We found no significant differences in baseline age (p = 0.845), disease duration (p = 0.258), mean logMAR BCVA (p = 0.765), visual functioning subscale score on the NEI‐VFQ (p = 0.616), ‘reading’ goal priority score on the PAI (p = 0.616), or person measure score on the OFQ (p = 0.546) between those who did and those who did not resume rehabilitation with the OrCam.

**Table 3:** Advantages and limitations of the OrCam reported by patients with retinitis pigmentosa or cone‐rod dystrophies.

Advantages	Limitations
(+) Text recognition in optimal light conditions (+) Portability (+) Hands‐free (+) Colour recognition (+) Barcode recognition (+) Bluetooth connectivity with earpieces	(−) Difficulties with text recognition in low light (−) Heavy and unbalanced on lightweight frames (−) Short battery life (−) No connectivity capabilities with your smartphone (−) Lack of desired features*

Remarks that were mentioned by at least five patients are listed.

* Example of features that were requested in this patient cohort included: assistance with navigation, voice activation, and internet connectivity.

## Discussion

The objective of this study was to investigate whether the OrCam could assist in performing daily activities and subsequently improve the quality of life in patients with RP or CRD. As visual function gradually declines in patients with IRDs, so does their ability to perform daily activities, which, in turn, results in reduced vision‐related quality of life (Chaumet‐Riffaud et al. 2017). As such, our cohort with severely visually impaired and blind patients with IRDs presented with markedly impaired of quality of life, as measured on the NEI‐VFQ.

When assessing the priority scores on the PAI, we found that the highest scores were found in the ‘mobility indoors within an unfamiliar location’ rehabilitation goal for patients with RP, whereas ‘reading’ and ‘personal administration’ were the most important rehabilitation goals in patients with CRD. These findings coincide with the different visual abilities present in patients with RP and CRD, with patients with RP most often facing challenges with mobility due to loss of peripheral vision, and patients with CRD experiencing difficulties with reading due to loss of central vision (Hamel 2006; Hamel 2007).

The Rasch‐calibrated OFQ revealed that the most difficult tasks were ‘reading a distant sign’, ‘reading a product label’ and ‘recognizing familiar faces within an unfamiliar environment’, as they required the highest visual ability of patients. These tasks share a common theme in that they all involve visual search behaviour, which is defined as the perceptual ability to actively scan the environment to locate the target of interest amongst other visual distractors (Timmis et al. 2017). Visual search requires input from central and peripheral vision, both of which are lost, to various degrees, in our patient cohort (Sullivan et al. 2008; Timmis et al. 2017).

After rehabilitation with the OrCam, significant improvements were seen in the ‘near activity’ subscale of the NEI‐VFQ. Similar results were found in a previous study with the OrCam, showing improvements in the ‘near vision’ subscale of the NEI‐VFQ in patients with end‐stage glaucoma (Waisbourd et al. 2019). As previous studies have demonstrated that the NEI‐VFQ suffers from multidimensionality, we also obtained Rasch estimates from visual functioning and socio‐emotional subscales (Stelmack et al. 2002; Pesudovs et al. 2010). Using this method, we found significant improvements in the visual functioning subscale, but no improvements in the socio‐emotional subscale at follow‐up. Significant improvements were also observed in the ‘reading’ goal on the PAI and the person measure score on the OFQ. These findings altogether suggest that the OrCam primarily improves reading abilities in patients with RP or CRD. The improvements after OrCam usage did not differ between phenotypes, which may be due to our limited sample size, impeding more in‐depth subgroup analysis. As suggested previously, it is possible that the level of visual acuity loss rather than visual field loss is important when selecting eligible patients for the OrCam (Waisbourd et al. 2019). Other features, such as facial and object recognition, were not tested by all patients during this relatively short follow‐up, and the impact of these features on the quality of life in patients with IRDs remains uncertain. For these features, patients are required to store the person or object into the memory of the OrCam, a process that could take more than several minutes for the current version of the OrCam for each person or object, which is potentially exhaustive and time‐consuming for severely visually impaired or blind patients over a study period of 5.2 weeks.

Most patients (85%) did not continue with rehabilitation, as they were in possession of other text‐to‐speech products, such as mobile applications with text recognition features (e.g. Seeing AI or the KNFB reader). These products share similar features with the OrCam, although, unlike the OrCam, most of these products often cannot be controlled hands‐free or through gesturing motions. However, these products are typically less expensive compared to the OrCam MyEye 2.0, which is currently available for approximately €3500 in Europe or $4500 in the United States. The higher costs of the OrCam may pose as an entry barrier for patients that wish to rehabilitate with the device. In order for the OrCam to be serviceable to more patients with IRDs, further improvements in the OrCam are needed. Examples of improvements suggested by patients include improved text recognition in low light conditions, connectivity capabilities with a smartphone and inclusion of additional features (e.g. navigation assistance), among others. Recently, a new version of the OrCam, the OrCam MyEye Pro, was released, which contains additional features such as smart reading and orientation features.

Several limitation and confounding factors were present in this study. This study included a relatively small sample size of patients with advanced stages of IRDs. Therefore, our findings may not be generalizable to other populations or to patients with higher visual abilities. Furthermore, this study only included one follow‐up assessment, as not all rehabilitation goals were met with OrCam rehabilitation, and withholding patients from receiving adequate rehabilitation for all their rehabilitation needs would be considered unethical (Wang et al. 2012). The possibility exists that patients overestimated or underestimated their functional changes with the OrCam, as they may not have accumulated enough real‐life experiences with the device within our relatively short study period. Additionally, as patients were aware of being observed, the possibility of a more positive response to rehabilitation with the OrCam to appease clinical researchers, that is, a Hawthorne effect, should not be disregarded (Lamoureux et al. 2007; McCambridge et al. 2014). Future studies that include longer follow‐up visits, different phenotypes, and a wider range of visual abilities would be invaluable to extend the current findings.

In conclusion, this study has provided a comprehensive overview of the OrCam MyEye 2.0 addressing both advantages and disadvantages of this device when prescribed to patients with RP or CRD. This knowledge may inform patients about the possibilities with the OrCam, whilst also setting realistic expectations, which, in turn, will facilitate the decision‐making process regarding the OrCam. The OrCam is a useful LVA to improve reading abilities in patients with RP or CRD. Further improvements in the OrCam may enhance its utility in the rehabilitation process of patients with RP or CRD.

## Supporting information


**Table S1**. Questionnaires used in this study and their included items.

**Table S2**. Baseline scores of the NEI‐VFQ, PAI and OFQ questionnaires.
