# Supplementary material for: Artificial vision: the effectiveness of the OrCam in patients with advanced inherited retinal dystrophies
Source: Acta Ophthalmol. 2021 Sep 26;100(4):e986–93. doi: 10.1111/aos.15001 (PMC9292690; doi:10.1111/aos.15001)
Supplement: Supplementary file 1 — Table S1. Questionnaires used in this study and their included items. [file AOS-100-e986-s001.docx]

**Supplemental Table 1**. Questionnaires used in this study and their included items.

| **NEI-VFQ**  **visual functioning subscale (original item #)*** | **NEI-VFQ**  **socio-emotional subscale**  **(original item #)*** | **Participation and Activity Inventory** | **OrCam Function Questionnaire**  **(original item #)*** |
| --- | --- | --- | --- |
| Eyesight (2) | Seeing how people react (11) | Reading | Reading a page from a book (1) |
| Reading ordinary print in newspapers (5) | Visiting people at their home, parties or restaurants (13) | Writing | Reading an e-mail (3) |
| Seeing well up close (6) | Accomplishing less (17) | Personal administration | Reading text from a distant sign, such as a street sign (4) |
| Finding something on a crowded shelf (7) | Staying at home most of the time (20) | Keeping time and following a schedule | Distinguishing different monetary bills (6) |
| Reading street signs or names of stores (8) | Having much less control (22) | Computer use | Distinguishing colors on a clothing piece (7) |
| Going down steps, stairs, or subs in dim light or at night (9) | Relying too much on what other people tell (23) | Mobility indoors at home | Recognizing objects, such as your keys or phone, at home (8) |
| Noticing objects off to the side while you are walking along (10) | Needing a lot of help from others (24) | Mobility indoors within an unfamiliar location | Recognizing a familiar product in the grocery store (9) |
| Picking out and matching your own clothes (12) | Worrying about doing something embarrassing (25) | Mobility outside | Reading a product label (11) |
| Going out to see movies, plays or sport events (14) | Receiving more help from others (A11a) | Public transportation | Recognizing familiar faces at home (12) |
| Read small print in a telephone book or medicine bottle (A3) | Being limited in things to do (A11b) | Grocery shopping | Recognizing familiar faces outdoors (13) |
| Checking accuracy of bills (A4) | Not leaving home alone (A13) | Recognition and communication | Telling time (14) |
| Shaving, styling and putting on make-up (A5) |  |  |  |
| Recognizing people across the room (A6) |  |  |  |
| Take part in sports or outdoor activities (A7) |  |  |  |
| See and enjoy programs on TV (A8) |  |  |  |

All questionnaires were administered in Dutch. Patients were instructed to answer all questions as if they were using all of their relevant visual aids. NEI-VFQ-25, National Eye Institute Visual Function Questionnaire-25. *These questionnaires were administered in full, but were subsequently re-engineered to fit Rasch analysis. Re-engineering of the NEI-VFQ was guided by previous studies (Stelmack et al. 2002; Pesudovs et al. 2010).
