# Supplementary material for: Artificial vision: the effectiveness of the OrCam in patients with advanced inherited retinal dystrophies
Source: Acta Ophthalmol. 2021 Sep 26;100(4):e986–93. doi: 10.1111/aos.15001 (PMC9292690; doi:10.1111/aos.15001)
Supplement: Supplementary file 2 — Table S2. Baseline scores of the NEI‐VFQ, PAI and OFQ questionnaires. [file AOS-100-e986-s002.docx]

**Supplemental Table 2.** Baseline scores of the NEI-VFQ, PAI and OFQ questionnaires.

|  | **Mean score ± SD** | | |  |
| --- | --- | --- | --- | --- |
|  | **Study group**  **(n = 20)** | **Retinitis pigmentosa (n = 9)** | **Cone-rod dystrophies**  **(n = 11)** | **p-value** |
| **NEI-VFQ subscale scores (max score = 100; higher score = better functioning)** | | | | |
| General health | 63.4 ± 14.5 | 64.7 ± 11.3 | 62.3 ± 17.2 | 0.718 |
| General vision | 31.5 ± 13.9 | 28.3 ± 13.2 | 34.1 ± 14.5 | 0.370 |
| Ocular pain | 79.4 ± 17.3 | 84.7 ± 18.5 | 75.0 ± 15.8 | 0.221 |
| Near activities | 37.5 ± 13.2 | 36.6 ± 17.7 | 38.3 ± 22.0 | 0.786 |
| Distance activities | 35.1 ± 18.3 | 38.0 ± 22.0 | 37.8 ± 15.4 | 0.543 |
| Social functioning | 53.3 ± 18.6 | 48.1 ± 23.5 | 57.6 ± 13.2 | 0.271 |
| Mental health | 63.0 ± 17.3 | 61.1 ± 18.7 | 65.5 ± 18.7 | 0.670 |
| Role limitation | 39.7 ± 19.6 | 37.5 ± 20.7 | 41.5 ± 19.4 | 0.674 |
| Dependency | 63.1 ± 18.0 | 66.7 ± 22.8 | 60.2 ± 13.5 | 0.441 |
| Color vision | 68.8 ± 28.0 | 69.4 ± 32.5 | 68.2 ± 25.2 | 0.923 |
| Peripheral vision | 43.8 ± 37.1 | 22.2 ± 23.2 | 61.4 ± 37.7 | 0.014 |
| Composite score | 51.6 ± 12.5 | 49.3 ± 13.9 | 53.5 ± 11.5 | 0.465 |
| **PAI priority scores (max score = 12; higher score = higher rehabilitation need)** | | | | |
| Reading | 6.3 ± 3.3 | 5.2 ± 3.0 | 7.1 ± 3.4 | 0.213 |
| Writing | 3.7 ± 4.0 | 4.6 ± 5.1 | 3.1 ± 2.5 | 0.430 |
| Personal administration | 6.6 ± 3.9 | 6.0 ± 4.0 | 7.0 ± 3.7 | 0.571 |
| Keeping time and following a schedule | 1.4 ± 2.5 | 0.7 ± 2.0 | 1.9 ± 2.8 | 0.276 |
| Computer use | 4.2 ± 2.9 | 3.7 ± 3.6 | 4.5 ± 2.0 | 0.544 |
| Mobility indoors at home | 2.1 ± 2.8 | 1.7 ± 2.6 | 2.5 ± 2.9 | 0.541 |
| Mobility indoors within an unfamiliar location | 5.8 ± 3.7 | 6.6 ± 4.8 | 5.1 ± 2.5 | 0.883 |
| Mobility outside | 4.2 ± 3.4 | 4.4 ± 4.0 | 4.0 ± 3.1 | 0.783 |
| Public transportation | 4.7 ± 3.1 | 5.2 ± 3.0 | 4.2 ± 3.2 | 0.488 |
| Grocery shopping | 5.9 ± 4.2 | 5.4 ± 4.0 | 5.4 ± 4.2 | 0.967 |
| Recognition and communication | 1.9 ± 2.5 | 2.3 ± 2.9 | 1.5 ± 2.1 | 0.483 |
| **OFQ difficulty scores (max score = 5; higher scores = more difficulty)** | | | | |
| Reading a page from a book | 3.5 ± 1.5 | 3.0 ± 1.6 | 4.0 ± 1.3 | 0.143 |
| Reading an e-mail | 2.0 ± 1.5 | 1.3 ± 0.7 | 2.5 ± 1.7 | 0.061 |
| Reading text from a distant sign, such as a street sign | 4.5 ± 0.8 | 4.4 ± 0.7 | 4.5 ± 0.8 | 0.977 |
| Distinguishing different monetary bills | 2.4 ± 1.2 | 2.6 ± 1.1 | 2.3 ± 1.3 | 0.622 |
| Distinguishing colors on a clothing piece | 3.2 ± 1.4 | 3.4 ± 1.4 | 2.9 ± 1.4 | 0.405 |
| Recognizing objects at home | 2.0 ± 1.0 | 1.9 ± 1.2 | 2.1 ± 0.9 | 0.673 |
| Recognizing products in the grocery store | 3.7 ± 0.9 | 3.9 ± 0.9 | 3.5 ± 0.9 | 0.423 |
| Reading a product label | 4.5 ± 0.5 | 4.4 ± 0.5 | 4.6 ± 0.5 | 0.418 |
| Recognizing familiar faces at home | 3.6 ± 1.5 | 3.4 ± 1.5 | 3.6 ± 1.5 | 0.780 |
| Recognizing familiar faces within an unfamiliar environment | 4.6 ± 0.8 | 4.7 ± 0.7 | 4.5 ± 0.7 | 0.731 |
| Telling time | 2.7 ± 1.5 | 2.3 ± 1.3 | 3.0 ± 1.6 | 0.333 |

NEI-VFQ-25, National Eye Institute Visual Function Questionnaire-25; OFQ, OrCam Function Questionnaire; PAI, Participation and Activity Inventory Questionnaire; SD, standard deviation.
